# Elevated NF-κB signaling in Asherman syndrome patients and animal models

**DOI:** 10.18632/oncotarget.14853

**Published:** 2017-01-27

**Authors:** Xiangzhen Wang, Nana Ma, Qiannan Sun, Chenlingzi Huang, Yanmei Liu, Xin Luo

**Affiliations:** ^1^ The First Affiliated Hospital of Jinan University, Guangzhou, Guangdong, China, 510630; ^2^ Nanshan Maternity and Child Healthcare Hospital of Shenzhen, Shenzhen, Guangdong, China, 518052; ^3^ The First Affiliated Hospital of Xinxiang medical college of Henan, Xinxiang, Henan, China, 453100; ^4^ Changzhi City People's Hospital of Shanxin Medical University Affiliated Hospital, Changzhi, Shanxi, China, 046000; ^5^ Huadu District, Guangzhou City People's Hospital, Guangzhou, Guangdong, China, 510630

**Keywords:** NF-κB, Asherman syndrome, intrauterine adhesion, pregnancy, uterine disease

## Abstract

Asherman syndrome (intrauterine adhesion) is often associated with menstrual abnormalities, infertility and recurrent miscarriage in female. Currently the molecular mechanism regulating the pathogenesis of Asherman syndrome is not known. Here we revealed that the inflammatory factor NF-κB expression is significantly elevated in the endometrial samples of Asherman syndrome patients. To further study the molecular mechanisms, we established an Asherman syndrome rat model and confirmed the important role of NF-κB in the pathogenesis of Asherman syndrome. In addition, our rat model provided direct evidence that intrauterine adhesion results in impaired pregnancy, supporting the clinical association between intrauterine adhesion and mis-regulated pregnancy. Our result identified NF-κB as a novel pathogenesis factor of Asherman syndrome and provided new insights for the prevention and treatment of intrauterine adhesions in Asherman syndrome patients.

## INTRODUCTION

Intrauterine adhesions (IUA) is a uterine disease with aberrant formation of adhesions within the uterus and/or cervix [1]. Patients with intrauterine adhesions are significantly associated with menstrual abnormalities such as amenorrhea, hypomenorrhea, menorrhagia and suffer from pelvic pain [1–3]. In addition, intrauterine adhesions could potentially prevent implantation of the blastocyst, impair the blood supply to the uterus and early fetus, and therefore result in the infertility or recurrent miscarriage in the Asherman syndrome patients [4–6].

Understanding the causes and related molecular mechanisms of intrauterine adhesions is essential for the prevention and treatment of Asherman syndrome. Intrauterine adhesions commonly occur after injury in the uterus cavity [7, 8]. It has been reported that post-infectious inflammation and inflammatory factors play important roles in the pathogenesis of Asherman syndrome [1, 8–10]. The nuclear factor-kappaB (NF-κB) transcription factor promotes the expression of intrauterine adhesion inflammatory factors and plays a central role in inflammatory diseases [11–15], however, whether NF-κB promotes the pathogenesis of Asherman syndrome remains unknown.

Here we investigated the role of NF-κB in intrauterine using Asherman syndrome patient samples and animal models. Our data revealed that the expression of NF-κB is significantly elevated in endometrial samples from intrauterine adhesion patients compared to normal endometrium controls. We then established an Asherman syndrome rat model and further confirmed the essential role of NF-κB in the pathogenesis of Asherman syndrome. Our result could potential benefit the prevention and therapy of intrauterine adhesions in Asherman syndrome patients.

## RESULTS

### Increased NF-κB expression in Asherman syndrome patient samples

**Figure 1:**
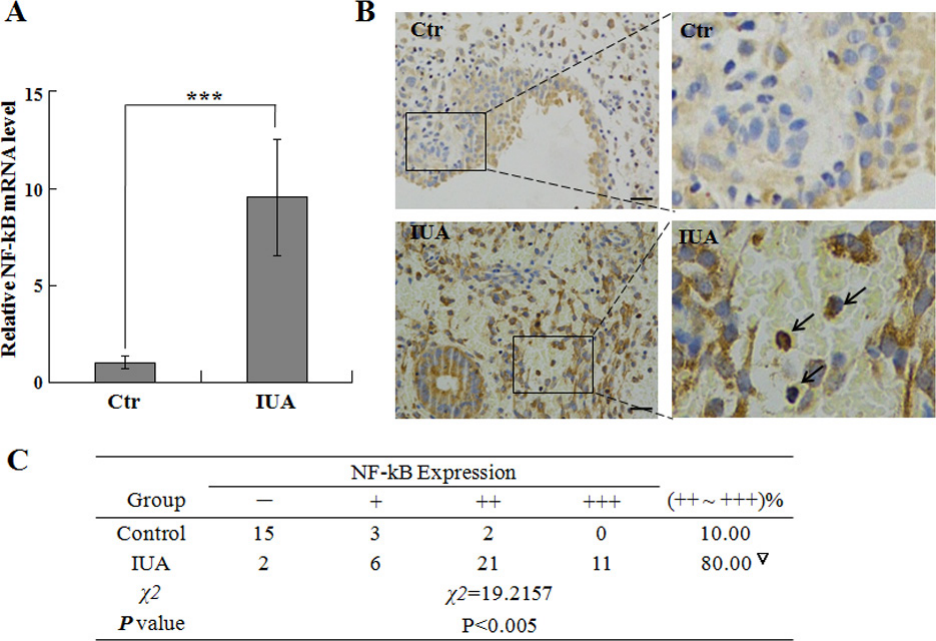
NF-κB expression in human Asherman syndrome patient samples Endometrial tissues were collected from Asherman syndrome patient and control individuals for analysis of NF-κB expression by quantitative PCR (**A**) and IHC staining (**B**). In Figure A, the relative expression of NF-κB was first normalized to internal control GAPDH, then the expression of NF-κB in the IUA samples were normalized to the NF-kB level in the controls (set as 1). Arrows indicate the representative NF-κB positive cells. Scale bar = 10 uM. The intensity and percentage of positive staining signal were summarized in Figure (**C**) ***P < 0.001.

To study the potential role of NF-κB in the pathogenesis of Asherman syndrome, we first determined the expression level of NF-κB in the intrauterine adhesion tissues from the Asherman syndrome patients. 40 Asherman syndrome patient samples and 20 normal control samples were used to study the mRNA and protein levels of NF-κB p65 subunit. As shown in Figure 1A, using Real-time Quantitative RT-PCR, we found that the expression of NF-κB mRNA is significantly higher in the endometrial samples from intrauterine adhesion patients compared to normal endometrial controls, suggesting the increased level of NF-κB function in the endometrial samples in Asherman syndrome patients.

Immunohistochemistry (IHC) staining is a powerful method to detect protein expression and localization in tissues [16], therefore we performed IHC staining to determine NF-κB protein level and location in Asherman syndrome patient and normal control samples. Our IHC data revealed that the NF-κB p65 expression in the normal endometrium control samples is low and mainly located in the cytoplasm (Figure 1B), while in the endometrial samples from intrauterine adhesion patients, the NF-κB staining is strong and mainly located in the nucleus (Figure 1B, arrows), suggesting the activation of NF-κB signaling in the patient IUA samples. Quantification and statistical analysis of NF-κB staining revealed that the NF-κB protein expression is significantly elevated in the IUA samples from Asherman syndrome patients (Figure 1C), further indicating the important roles of NF-κB in the pathogenesis of Asherman syndrome.

### Establish an Asherman syndrome animal model with phenol mucilage

**Figure 2:**
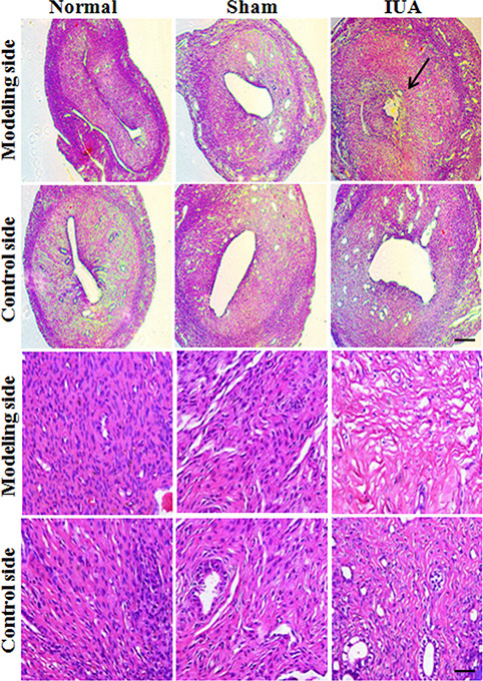
HE staining revealed the abnormal morphology in Asherman syndrome rats 60 rats were randomly separated into three different groups: IUA group (30 rats for phenol mucilage treatment), sham group (15 rats with mock treatment) and normal group (15 rats with no treatment). For each rat, only one side of the uteruses was treated with phenol mucilage (modeling side), the other untreated side was defined as control side. The uteruses from three groups were collected for HE staining 10 days after treatment. Arrow indicates the intrauterine adhesion. Upper panels scale bar = 100 uM, lower panels scale bar = 10 uM.

**Figure 3:**
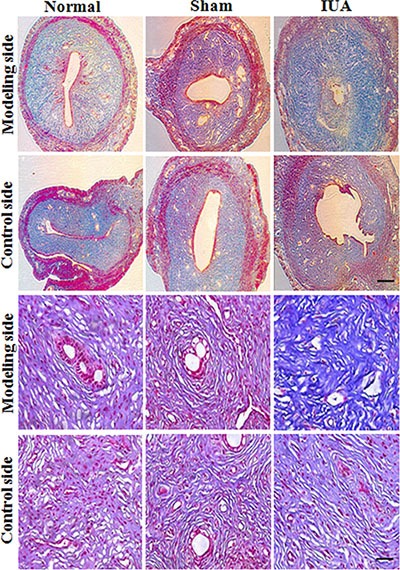
Masson staining revealed the abnormal morphology in Asherman syndrome rats Rats were randomly separated into three different groups: IUA group (phenol mucilage treatment), sham group (mock treatment) and normal group (no treatment). Only one side of the uteruses was treated with phenol mucilage (modeling side), the other untreated side was defined as control side. 10 days after treatments, both sides (modeling side and control site) of the uteruses were collected for Masson staining. Upper panels scale bar = 100 uM, lower panels scale bar = 10 uM.

To provide direct evidence of NF-κB in regulating pathogenesis of Asherman syndrome, we established an intrauterine adhesion rat model using phenol mucilage. Phenol mucilage was widely used for female sterilization by inducing the formation of intrauterine adhesion [17]. The phenol mucilage method is better than other methods such as mechanical injury to induce IUA because it's easier to control the levels of damage using liquid phenol mucilage. As shown in Figure 2, HE staining of the uteruses revealed the abnormal morphology in the modeling uteruses from the IUA group. The uterine cavity was small and filled with hyperplastic tissues (Figure 2, arrow). The endometrial structure was irregular, the macrophages and plasma cells decreased and were replaced by large number of fibroblast (Figure 2, lower panel), suggesting the mis-regulated inflammation and repair process during the pathogenesis of IUA. We further performed Masson staining and found that the modeling uteruses from the IUA group had increased blue staining cells, suggesting severe fibrosis occurred in the modeling site of the IUA group uteruses (Figure 3). Overall, the above results revealed the successful establishment of Asherman syndrome rat model by phenol mucilage.

**Figure 4:**
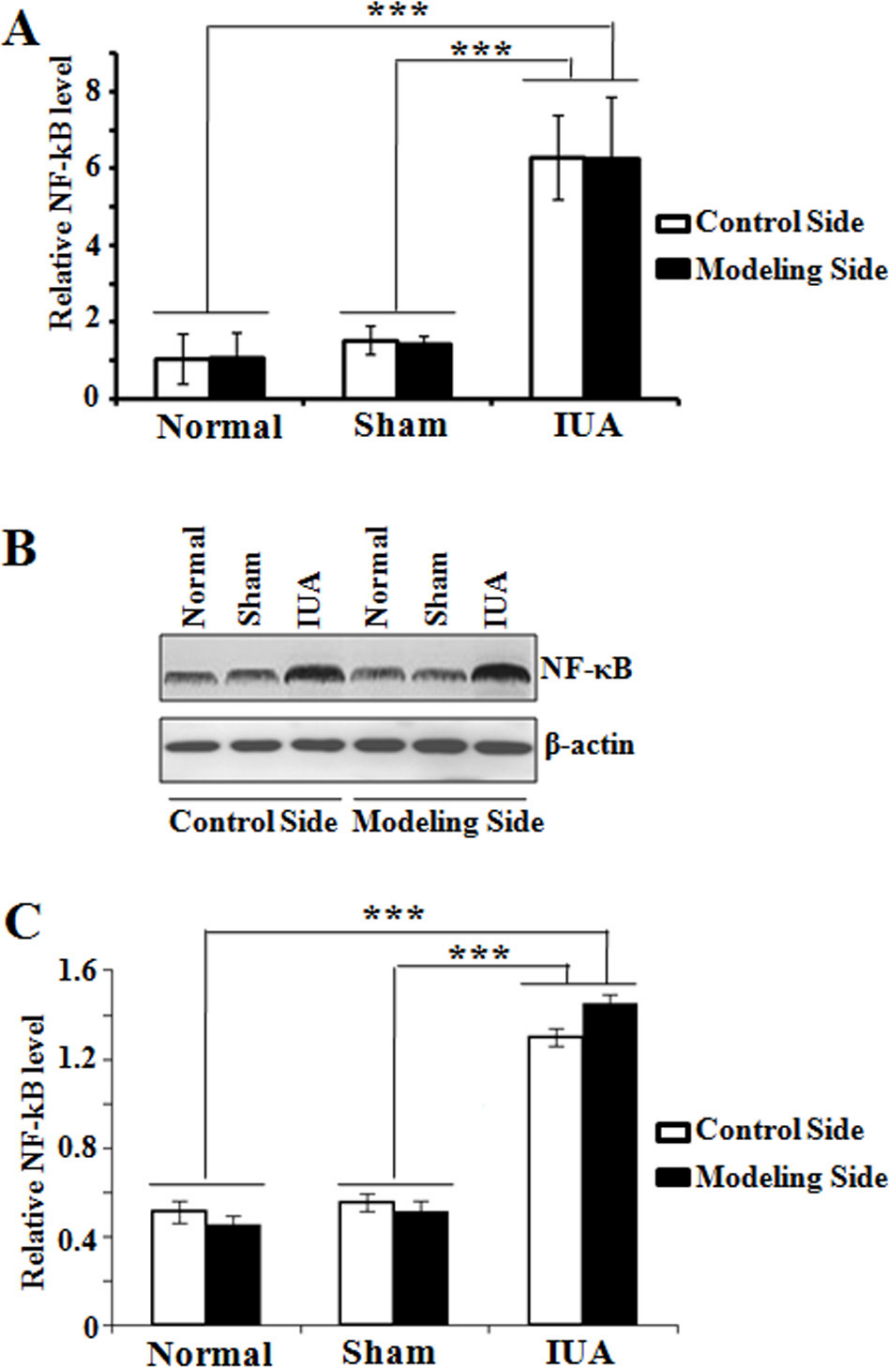
Determination of NF-κB expression in Asherman syndrome rats Endometrial tissues were collected both sides of the uteruses from three different rat groups. (**A**) quantitative RT-PCR analysis of NF-κB mRNA expression; Normal: *n* = 7; Sham: *n* = 7; IUA: *n* = 15. ***p = 0.001. (**B**) Western blotting analysis of NF-κB protein expression; (**C**) Quantification of NF-κB protein in B. *n* = 5, ***p = 0.001.

### Elevated NF-κB expression in Asherman syndrome rat model

**Figure 5:**
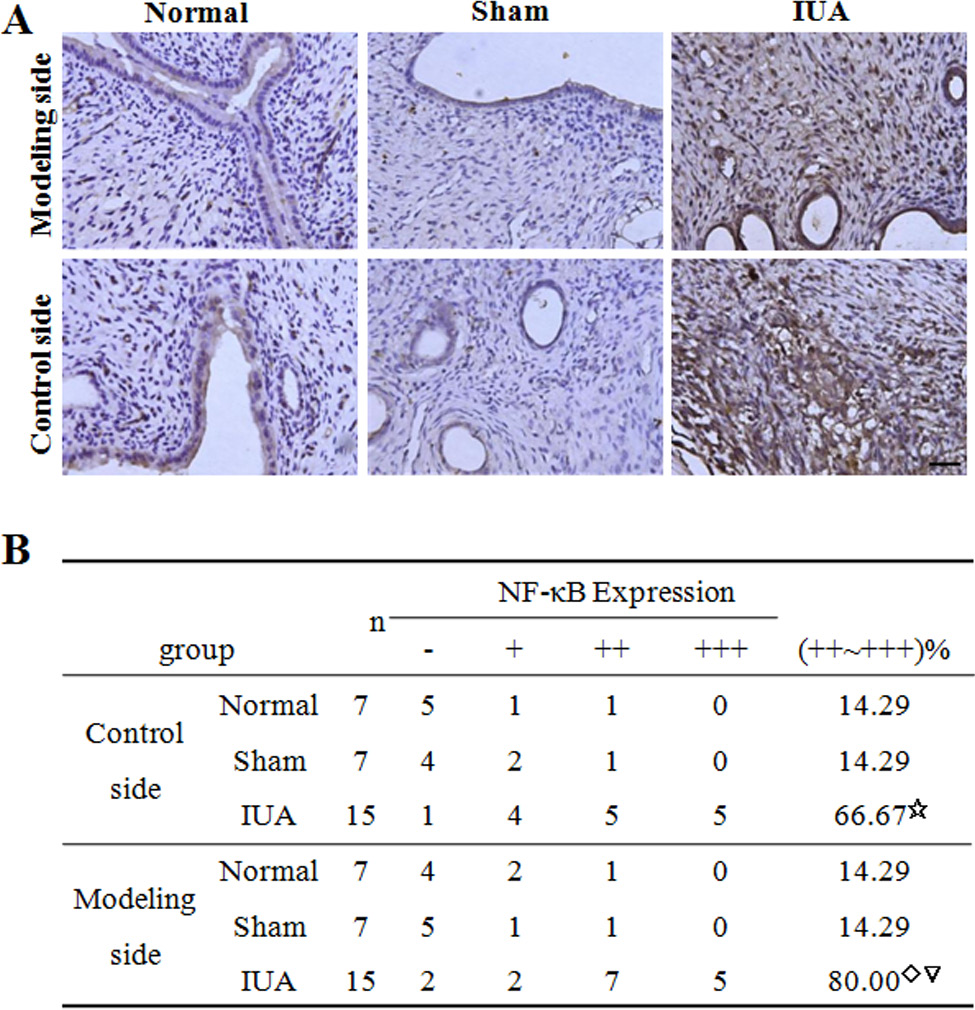
IHC staining of NF-κB in Asherman syndrome rats Endometrial tissues were collected both sides of the uteruses from three different rat groups. (**A**) IHC staining of NF-κB in different samples. Scale bar = 10 uM. (**B**) Summarization of intensity and percentage of positive staining signal in A. ^☆^Comparison of IUA control side to Normal, Sham control sides, *F* = 17.1, *P* = 0.02; ^◇^Comparison of IUA modeling side to Normal, Sham modeling sides, *F* = 18.3, *P* = 0.01; ^▽^Comparison between control and modeling sides in IUA group, *t* = 0.325, *P* = 0.441.

With the Asherman syndrome rat model, we next determined the expression of NF-κB in modeling and control sides of uteruses from the normal, sham, and IUA group rats. We found that NF-κB mRNA expression significantly increased (6 fold) in both modeling and control sides of uteruses from the IUA group rats compared to those from the normal and sham groups. And there was no significant difference of NF-κB mRNA expression in the uteruses between the normal and sham groups (Figure 4A). Western blotting using anti-NF-κB p65 antibody also confirmed the up-regulation of NF-κB p65 expression in the uteruses from the IUA group (Figure 4B, 4C). We further performed IHC staining of the uteruses from three groups and revealed that the uteruses from IUA group showed strong positive NF-κB staining (Figure 5A). The NF-κB mainly located in the nucleus of the endometrial cells and some of the glandular epithelial cells and endometrial basal cells, suggesting that activation of NF-κB signaling in those cells. Quantification of the staining revealed a significant increase of NF-κB in the uteruses from the IUA group compared to the normal and sham groups in both control side and modeling side (Figure 5B). Interestingly, there was no significant difference of NF-κB expression between the modeling side and control side in the IUA group, which might be explained by the internal circulation within the two sides of uterus. Overall, the above data further confirmed the critical role of NF-κB in the in pathogenesis of Asherman Syndrome.

**Figure 6:**
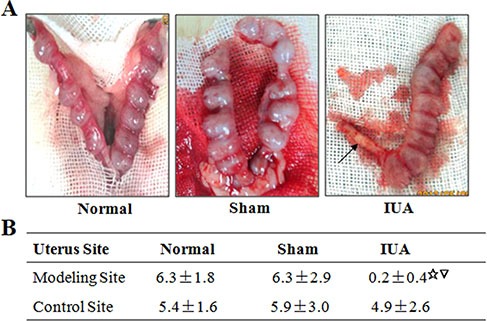
Intrauterine adhesion impaired pregnancy in Asherman syndrome rats Pregnant rats of three groups were sacrificed to study the number of embryos in the uteruses. (**A**) Representative pictures of the isolated uteruses from three groups of rats. Arrow indicates the intrauterine adhesion. (**B**) Summary of embryo numbers in both side of uteruses from three rat groups. Normal: *n* = 8; Sham: *n* = 8; IUA: *n* = 15. ^☆^Comparison between three rat groups, *P* = 0.001; ^▽^Comparison between control and modeling sides in IUA group, *P* = 0.001.

### Impaired pregnancy in the Asherman syndrome rat model

Intrauterine adhesion is associated with infertility or recurrent miscarriage in the Asherman syndrome patients [4–6]. However, there is no direct evidence that intrauterine adhesion causes impaired pregnancy. Using the Asherman syndrome rat model, we determined the effect of intrauterine adhesion in pregnancy outcomes in our three experimental groups of rats. We found that the normal and sham group rat can achieve normal pregnancy, while the rats in the IUA group had significant less number of embryos in the modeling side uteruses compared to those from normal and sham groups (Figure 6A, 6B). The control side uteruses of the IUA rats had fewer embryos but the difference is not statistically significant (Figure 6A, 6B). Overall, our data provided direct evidence that intrauterine adhesion results in pregnancy failure in the Asherman syndrome rats.

## DISCUSSION

Intrauterine adhesion is often caused by infection or injury related inflammation. Inflammation is a cellular process that coordinates gene expression and controls the tissue microenvironment [18–20]. Inflammatory factors especially cytokines such as TGF-β, TNF-α, IL-1 and IL-18 are frequently elevated in intrauterine adhesions and actively promote the pathogenesis of Asherman syndrome [21, 22]. NF-κB pathway is a critical regulatory pathway that controls cytokine production and cell survival. NF-κB is essential for the immune response and is found to actively promote the pathogenesis of many inflammatory diseases such as inflammatory bowel disease, arthritis and others [12, 23]. Here we found that NF-κB mRNA and protein levels are elevated in intrauterine adhesion tissues in both Asherman syndrome patients and rats. IHC study further confirmed the activation of NF-κB signaling in intrauterine adhesions. NF-κB closely cross-talks with intrauterine adhesion pathogenesis factors such as TGF-β, TNF-α, IL-1 and IL-18 [24–27]. Our discovery that NF-κB is significantly up-regulated and activated in intrauterine adhesion tissues identified NF-κB as a novel pathogenesis factor of Asherman Syndrome.

Establishment of Asherman syndrome animal model is essential for the study of Asherman syndrome phenotype, pathogenesis mechanisms and the development of effective therapeutic strategies for the treatment of Asherman syndrome. Here we successfully induced the formation of intrauterine adhesion in rats using the phenol mucilage method, which has better consistency compared to the IUA models induced by other methods such as mechanical injury. The Asherman syndrome rats have small uterine cavities, abnormal hyperplastic tissues, decreased macrophages and plasma cells, and severe fibrosis in the uteruses, representing the typical phenotype of intrauterine adhesions. We further revealed that the intrauterine adhesions significantly reduced the fetus numbers, providing direct evidence that intrauterine adhesions cause impaired pregnancy in Asherman syndrome rats.

In summary, our data provided direct functional link between intrauterine adhesions and pregnancy defects using Asherman syndrome animal model. In addition, we identified NF-κB as a novel pathogenesis factor of intrauterine adhesions in Asherman syndrome patients and rats. NF-κB could potentially serve as diagnostic marker and therapeutic target for the prediction and treatment of intrauterine adhesions.

## MATERIALS AND METHODS

### Patient information

Intrauterine adhesion and control tissues were collected between May 2014 and August 2014 from Nanshan Maternity & Child Healthcare Hospital of Shenzhen, Guangdong, China. 40 intrauterine adhesion tissues from Asherman syndrome patients and 20 normal endometrium tissues from secondary infertility control individuals were collected during hysteroscopy. There was no significant difference in age between the Asherman syndrome and control group. Written informed consents were obtained from all patients and the study protocols were approved by the Institutional Ethic Committee of the Hospital.

### Establish an Asherman syndrome animal model

The formation of intrauterine adhesion in rats was induced using phenol mucilage. 60 rats were randomly separated into three different groups: IUA group (30 rats for phenol mucilage treatment), sham group (15 rats with mock treatment) and normal group (15 rats with no treatment). For each rat, only one side of the uteruses was treated with 0.04ml phenol mucilage (modeling side), the other untreated side was defined as control side. 10 days after treatments, both sides (modeling side and control side) of the uteruses from three groups were collected for the analysis of Asherman syndrome related phenotypes.

### RNA isolation and quantitative real-time PCR analysis

Total RNA samples were isolated from tissues using Trizol as previously described [28]. Briefly, the frozen tissues were homogenized and then lyzed with Trizol reagent for the isolation of RNA. Remaining genome DNA was removed by gDNA Eraser treatment. 2 ug of total RNA was used for reverse transcription and subsequent quantitative Real-Time PCR. GAPDH was used as internal control to calculate the relative expression of NF-κB in different tissues. The primer sequences are: NF-κB F: GGTTACGGGAGATGTGAAGATG; NF-κB R: AAGGTGGATGATGGCTAAGTGT. GAPDH F: ACA GCAACAGGGTGGTGGAC; GAPDH R: TTTGAGGG TGCAGCGAACTT. Data were presented as mean ± sem from three independent experiments.

### IHC staining and scoring standard

IHC was performed as previously described using anti-NF-κB p65 antibody (Cell Signaling Technology #8242) [16]. To determine the relative expression of signal NF-κB in different tissues, both the percentage of positive cells and the intensity of the staining signal were evaluated to calculate the expression scores. 4 fields were randomly selected for each slide to determine its percentage score and intensity score. The percentage score was determined as followed: ≤ 10% positive, 0 point; 11% ~ 25% positive, 1 point; 25% ~ 50% positive, 2 points; > 50% positive, 3 points. Similarly, the signals on different fields were scored from 0 point to 3 points depending on its intensity. The expression score was calculated by multiplying the percentage score and intensity score. Then the expression levels of NF-κB in different tissues were separated into four different levels based on the expression scores: negative (−, 0–1 point); weak positive (+, 2–4 points); positive (++, 5–6 points); strong positive (+++, > 6 points).

### HE staining and masson staining

HE staining and Masson staining were performed as previously described [29, 30]. For HE staining, the slides were first deparaffinized and rehydrated, and then stained with hematoxalin and eosin. For Masson staining, the deparaffinized and rehydrated slides were incubated with Masson staining mixture for 5 minutes, then stained with Phosphomolybdic acid-aniline blue solution for 6.5 minutes.

### Western blot

Western blotting was performed as previously described [31, 32]. Briefly, tissues were homogenized and lyzed in protein lysis buffer on ice for 30 minutes. The collected protein samples were separated by SDS-PAGE, transferred to PVDF membrane and then blotted with indicated antibodies (anti-NF-κB p65 antibody: Cell Signaling Technology #8242; anti-β-actin: BD Transduction Laboratories^™^ 612656).

### Statistical analysis

All measurement data were presented as mean ± sem. One-way ANOVA was used to analyze the data from different groups. The data were analyzed by SPSS for Windows 17.0 software package. *P* < 0.05 was defined as statistically significant.
